# Seasonal Characteristics of the Chemical Composition of Fine Particles in Residences of Nanjing, China

**DOI:** 10.3390/ijerph16061066

**Published:** 2019-03-25

**Authors:** Guozhi Cao, Jun Bi, Zongwei Ma, Zhijuan Shao, Jinnan Wang

**Affiliations:** 1 State Key Laboratory of Pollution Control & Resource Reuse, School of the Environment, Nanjing University, Nanjing 210023, China; caogz@caep.org.cn (G.C.); jbi@nju.edu.cn (J.B.); wangjn@caep.org.cn (J.W.); 2 State Environmental Protection Key Laboratory of Environmental Planning and Policy Simulation, Chinese Academy for Environmental Planning, Beijing 100012, China; 3 Jiangsu Collaborative Innovation Center of Atmospheric Environment and Equipment Technology, Nanjing University of Information Science & Technology, Nanjing 210044, China

**Keywords:** metallic elements, water-soluble ions, seasonal variation, indoor air quality, health risk assessment

## Abstract

Indoor fine particulate matter (PM_2.5_) and its chemical composition is important for human exposure as people spend most of their time indoors. However, few studies have investigated the multiseasonal characteristics of indoor PM_2.5_ and its chemical composition in China. In this study, the chemical composition of PM_2.5_ samples in residences was analyzed over four seasons in Nanjing, China. Indoor water-soluble ions exhibited similar seasonal variations (winter > autumn > summer > spring) to those from outdoors (winter > autumn > spring > summer) except in summer. Whereas, indoor metallic elements exhibited a different seasonal pattern from that of outdoors. The highest concentrations of indoor metallic elements were observed in summer when the outdoor concentrations were low. The different seasonal variations of the chemical composition between indoor and outdoor PM_2.5_ indicated that people should consider both indoor and outdoor sources to reduce their exposure to air pollutants in different seasons. The carcinogenic risks for metallic elements were within the acceptable levels, while manganese (Mn) was found to have potential noncarcinogenic risk to humans. More attention should be paid to the pollution of Mn in the study area in the future. Moreover, the cumulative effect of noncarcinogenic PM_2.5_-bound elements should not be ignored.

## 1. Introduction

With the development of the economy and population, developing countries, such as China, are now facing increasing air pollution. In 2017, the air quality in more than 70% of Chinese cities exceeded the National Air Quality Standard, with ambient fine particulate matter (PM_2.5_) as the dominant air pollutant [1]. PM_2.5_ had been identified as one of the leading risk factors for global disease [2]. Exposure to PM_2.5_ has been shown to have associations with adverse health effects in epidemiological studies, such as cardiovascular and respiratory diseases, cancer, and preterm birth [3,4,5,6]. The degree of PM_2.5_-related toxicity mainly depends on its chemical components, including water-soluble inorganic ions, metallic elements, and organic and elemental carbon [7]. Water-soluble inorganic ions, such as sodium (Na^+^), potassium (K^+^), calcium (Ca^2+^), ammonia (NH_4_^+^), nitrate (NO_3_^−^), chloride (Cl^−^), and sulfate (SO_4_^2−^), were found to be the main components of PM_2.5_ and act as surface active reagents, which increase the toxicity of noxious organic substances [8]. The other main components that determine the toxicity of PM_2.5_ are metallic elements, such as lead (Pb), manganese (Mn), copper (Cu), chromium (Cr), arsenic (As), and cadmium (Cd), which can cause detrimental health impacts to human respiratory, cardiovascular, and nervous systems [9,10,11]. Understanding the chemical characteristics of PM_2.5_ concentrations is necessary to reduce its adverse impact on human health.

Many studies, which focused on the concentrations, species, spatial-temporal variations, and source identification, have investigated the components of ambient PM_2.5_ in Chinese cities and provided suggestions for ambient PM_2.5_ reduction [12,13,14]. Ambient PM_2.5_ component data have been widely used in human exposure and health risk assessments [15,16,17]. However, concentrations of ambient PM_2.5_ and its components cannot completely represent human exposure concentrations as people spend approximately 80%–90% of their times indoors, such as in residences, offices, vehicles, and classrooms [18,19,20]. Indoor PM_2.5_ can arise from both outdoor sources (e.g., traffic, industry and heating emissions) and indoor sources (e.g., cooking, smoking, and resuspension during indoor activities) [21]. Therefore, the concentrations of indoor PM_2.5_ and its chemical components may differ from the outdoors, which may lead to a bias in health effect assessments [22,23,24]. In this context, research on indoor PM_2.5_ and its chemical components is helpful for improving the accuracy of exposure and health effect estimates.

Due to the importance of indoor exposure, studies of indoor PM_2.5_ and its effect on human health have received more and more attention in recent years. Many related studies have been performed to investigate indoor PM_2.5_ in China [25,26]. The concentration of PM_2.5_ and its chemical components were investigated in some studies, and differences between indoors and outdoors were observed, depending on the seasons [27,28,29], indoor activities [27,30], ventilation systems [31], cooking fuels [26], etc. However, most of the studies were conducted in a single season or during specific periods (e.g., heating seasons or heavy haze-fog episodes) [32,33,34]. Few studies investigated the seasonal variation of chemical compositions of PM_2.5_ in a single family [29], which may not be representative. To our knowledge, little is known about the multiseasonal characteristics of the chemical components of PM_2.5_ in multiple residences in China. 

To fill this gap in the knowledge, we conducted a study in multiple residences of Nanjing, China, to characterize the concentration and chemical composition (water-soluble inorganic ion and metallic element content) in indoor and outdoor PM_2.5_ samples over four seasons. The objectives of the study were to examine the multiseasonal characteristics of chemical-species in indoor PM_2.5_ and explore the indoor and outdoor difference. The health risk of toxic elements in PM_2.5_ was assessed for adults and children. The results of this study will provide important implications for improving indoor air quality in residences and reduce human exposure and health impacts.

## 2. Materials and Methods

### 2.1. Study Area

Nanjing, a city located in the eastern part of China (N 31°14′~32°37′, E 118°22′~119°14′), is an important central city in the Yangtze River Delta region. The climate is subtropical, with hot summers, cold winters, and comfortable transitional seasons (spring and autumn). The city of Nanjing has a population of over 8,200,000. The air pollution in Nanjing is serious, with PM_2.5_ as one of the primary pollutants. In 2016, the ambient air quality index was rated excellent or good for 66.1% with an annual PM_2.5_ concentration of 47.9 μg/m^3^ [35], which is much higher than the annual mean standard (35 μg/m^3^) of the National Ambient Air Quality Standards of China.

### 2.2. Sampling Sites

The study was conducted from March 2016 to February 2017. A total of 124 homes participated in the study, with 34 homes in spring, 35 in summer, 20 in autumn, and 35 in winter. The details of the process of home recruitment were described by Shao et al. [36]. All the homes that participated in this study were apartments in urban areas with no obvious industrial sources around. The details of the home characteristics are shown in Table 1. A brief description of the sampling approach is included as follows. Indoor and outdoor PM_2.5_ concentrations were measured simultaneously by personal exposure micromonitors (MicroPEM; RTI International, Research Triangle Park, NC, USA). The indoor monitors were placed in the living room of each family, approximately 1.5 m above the ground, while the outdoor measurements were taken on the open balcony outside of the home. Two continuous 24-hour indoor and outdoor measurements were taken simultaneously at each home. The real-time concentrations of PM_2.5_ were recorded every 10 s by the MicroPEM. The poly tetra fluoroethylene PTFE filters (25 mm, 3.0 mm pore size, PALL, NewYork, NY, USA) in the MicroPEMs were replaced by our researchers every 24 h. The two filters obtained from each home were then taken to the laboratory of Nanjing University for water-soluble inorganic ion and metallic element analysis.

### 2.3. Water-Soluble Ions Analysis

One of the two PTFE filters obtained from each sampling home was used to analyze the concentrations of water-soluble inorganic ions, including five cations (Na^+^, K^+^, Ca^2+^, Mg^2+^, NH_4_^+^) and three anions (NO_3_^−^, Cl^−^, SO_4_^2−^). Each filter was cut into four slices and put in a centrifuge tube. 10 mL of ultra-pure water (specific resistance ≥ 18 Ω cm) was added to it. Then, the water-soluble ions were extracted by ultrasound for 30 min in a lower temperature. The extractions were filtered through a micro-porous membrane (pore size, 0.45 μm; diameter, 25 mm), and transferred to clean plastic bottles. The filtrates were stored in the refrigerator at 4 °C until analysis. Blank filters were also analyzed in the same way as samples, and the values were subtracted from the sample results. 

An ion chromatography instrument (ICS-1000, Dionex, Sunnyvale, CA, USA) operating at a flow rate of 1.0 mL/min was used to analyze the eight inorganic ions in PM_2.5_. The standard solution for water-soluble inorganic ions was purchased from the Research Center of China National Standard Reference Materials. The standard solution was diluted by ultra-pure water to mass concentrations of 0.00, 0.20, 0.50, 1.00, 2.00, 5.00, and 10.00 mg/L to obtain standard curves. The concentrations of water-soluble inorganic ions in the solution were calculated using the standard curves.

### 2.4. Metallic Elements Analysis

For the metallic element analysis, the filters were placed in a Teflon digestion tube. 10 mL of 1:1 HNO_3_ (spectral purity) was added into the tube and digested with a digestion system at a temperature of 106 ± 0.5 °C. When the solution remained at approximately 1 mL, another 4 mL of 1:1 HNO_3_ was added into the tubes after cooling. The digestion was then continued until there was approximately 1 mL solution remaining in the tube. Finally, 2 mL of 30% H_2_O_2_ (spectral purity) was added after cooling and digestion was completed until the remaining solution was 0.50–1.0 mL. The remaining solution was diluted with ultrapure water (specific resistance ≥ 18 Ω cm) to 10.0 mL and then filtered through a micro-porous membrane (pore size, 0.45 μm; diameter, 25 mm). The filtrate was stored at 4 °C until the metallic element analysis.

Metallic elements from the filtrate, including Pb, Mn, Cu, Cr, As, and Cd, were analyzed by inductively coupled plasma mass spectrometry (ICP-MS, NexION300×, PerkinElmer, Waltham, MA, USA) based on the United States Environmental Protection Agency (USEPA) 3050B method. A standard solution (ICP-MS#5183-4688) with a concentration of 10 mg/L was used. The solution was diluted with 0.1 M HNO_3_ to standard concentrations of 0.00, 0.50, 1.00, 2.00, 5.00, 10.00, and 20.00 μg/L to obtain the quantitative calibration curve. Then, 0.75 mL of filtrate of each sample was diluted to a 1.5 mL solution with 0.1 M HNO_3_ and the diluted filtrate was analyzed by ICP-MS to obtain the concentrations of the metallic elements in the solution.

### 2.5. Data Analysis

The water-soluble inorganic ion concentrations, measured by ICS, and metallic elements, measured by ICP-MS, were all corresponding concentrations in the filtrates. The chemical component concentrations of PM_2.5_ in the air were then converted as follows [28]:

(1) $$C_{i,a}=(C_{i,s}-C_{b})\;\times \;Vol_{s}÷\left(F_{a}\;\times \;T_{a}\right)$$

where $C_{i,a}$ is the mass concentration of ambient water-soluble ions or elements, μg/m^3^; $C_{i,s}$ is the corresponding ion and element mass concentration in the solution, μg/L;$\;C_{b}$ is the mass concentration of the blank filter in the solution, μg/L; $Vol_{s}$ is the volumne of the solution, L; $F_{a}$ is the flow rate of the MicroPEM, 0.0005 m^3^/min; and $T_{a}$ is the sampling time of the filter, min.

The relationship of indoor and outdoor PM_2.5_ components was studied. The indoor to outdoor (I/O) ratios of PM_2.5_ components were calculated as follows:

(2) $$I/O_{i}\;=\frac{C_{in\;i}}{C_{out\;i}}$$

where $C_{in\;i}$ is the concentration of chemical component *i* in indoor PM_2.5_; $C_{out\;i}$ is the corresponding concentration of chemical component *i* in outdoor PM_2.5_.

The indoor/outdoor correlation coefficient of PM_2.5_ chemical compositions was evaluated by the Spearman correlation analysis. The statistical analyses of indoor and outdoor PM_2.5_ concentrations and chemical components were performed using SPSS 18.0. 

### 2.6. Health Risk Assessment

The health risk assessment of PM_2.5_-bound metallic elements by inhalational exposure pathways for adults and children was conducted based on the method provided by tUSEPA [37]:

(3) $$EC_{i}=\left(CA_{i}\;\times \;\mathrm{ET}\;\times \;\mathrm{EF}\;\times \;\mathrm{ED}\right)/\mathrm{AT}$$

where EC*_i_* is the exposure concentration of metallic element *i* (ng/m^3^); CA*_i_* is the concentration of metallic element *i* in the air (ng/m^3^).; in this study, the upper bounds of the 95% confidence interval for the mean concentration of metallic elements *i* (C_UCL_ *_i_*), regarded as an estimate of the “reasonable maximum exposure,” were referred to as “CA*_i_*” [29]; ET is the exposure time (hours/day); EF is the exposure frequency (days/year); ED is the exposure duration (24 years for adults and 6 years for children); AT is the average time, for carcinogens (AT = lifetime in years × 365 days/year × 24 h/day) and for noncarcinogens (AT = ED in years × 365 days/year × 24 h/day).

The health risk assessment for metallic elements was quantified for noncarcinogenic and carcinogenic effects. The noncarcinogenic risk was evaluated by the hazard quotient (HQ), while the carcinogenic risk was evaluated by the excess cancer risk (CR) using the following equation:

(4) $${\mathrm{HQ}}_{i}=\frac{EC_{i}}{RfC_{i}}\times \;10^{-6}$$

(5) $$\mathrm{HI}=\sum^{}{\mathrm{HQ}}_{i}$$

where HQ_i_ is the hazard quotient of metal elements i; RfC_i_ is the inhalation reference concentration for the non-carcinogenic element i (mg/m^3^); and HI (hazard index) is the sum of HQ. An HI ≤ 1 indicates no significant risk of noncarcinogenic health effects, whereas an HI > 1 indicates the possibility of a noncarcinogenic health effect [37].

(6) $${\mathrm{CR}}_{i}=EC_{i}\;\times \;IUR_{i}\;\times \;10^{-3}$$

(7) $$\mathrm{CR}=\sum^{}{\mathrm{CR}}_{i}$$

where CR_i_ is the cancer risk of the metal elements; IUR_i_ is the inhalation unit risk for carcinogenic element i (μg/m^3^)^−1^; and CR is the sum of CR_i_. When the CR is >10^−4^, it indicates a serious risk of cancer, while a CR value between 1.0 × 10^−4^ and 1.0 × 10^−6^ is acceptable. When CR is <10^−6^, it indicates that the risk is minimum.

The RfC and IUR for selected metallic elements are cited from the USEPA website in the screening level (RSL) tables shown in Table 2 [38]. For the metallic element, Cr, it is important to notice that Cr in the environment is usually composed of Cr^6+^ and Cr^3+^. The concentrations of Cr measured in this study are the total concentrations of Cr^6+^ and Cr ^3+^. Cr^6+^ is classified as a human carcinogen, while Cr^3+^ has no carcinogenic effects. The ratio of Cr^6+^ to Cr^3+^ has been reported to be 1:6. Therefore, the concentrations of Cr^6+^ in this study were assumed to be 1/7 of the total Cr to calculate the health risk [39,40].

## 3. Results and Discussion

### 3.1. Seasonal Variations of PM_2.5_ and Chemical Composition

#### 3.1.1. PM_2.5_ Concentrations

During the sampling period, the average concentrations of PM_2.5_ in indoor and outdoor environments were 46.86 ± 21.75 μg/m^3^ and 51.44 ± 29.15 μg/m^3^, respectively. Generally, the indoor PM_2.5_ concentrations were lower than those from outdoors. Seasonal variability of both indoor and outdoor PM_2.5_ concentrations was observed in this study with the highest concentrations occurring in winter and the lowest concentrations occurring in summer. Detailed information about the seasonal variations of indoor/outdoor PM_2.5_ and influencing factors were discussed in our previous study [36].

#### 3.1.2. Water-Soluble Ions in PM_2.5_

**Figure 1:**
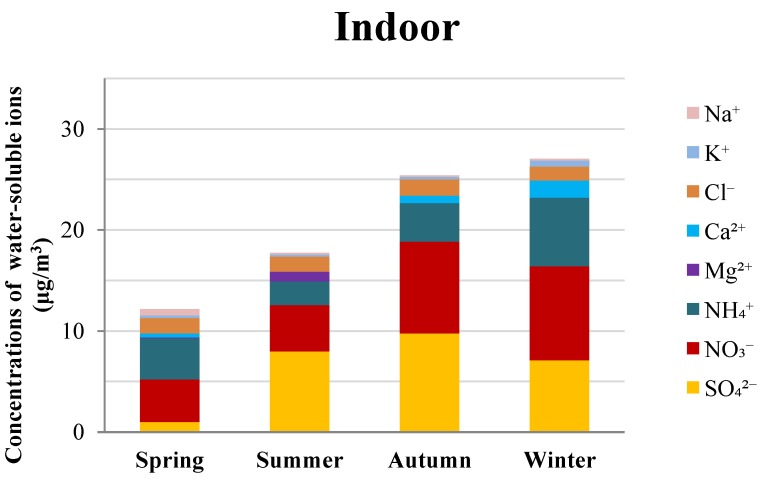

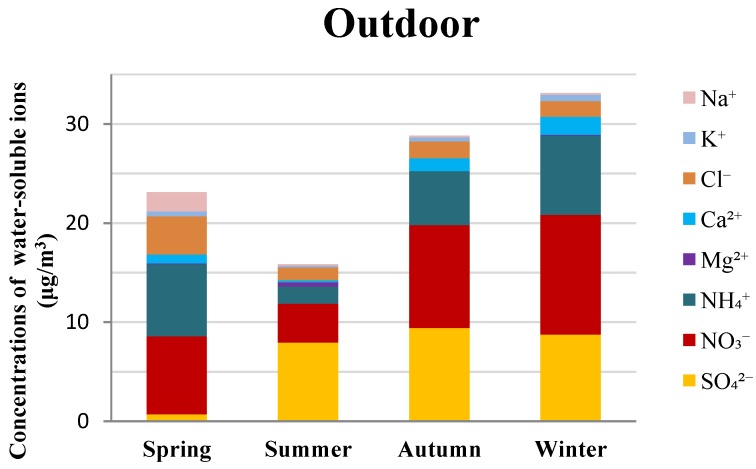
Seasonal variations of water-soluble ions in indoor and outdoor PM_2.5_.

The mass concentrations of inorganic water-soluble ions in PM_2.5_ presented distinctly seasonal distribution features as shown in Figure 1. For the outdoor PM_2.5_, the order of the total water-soluble ion concentration (Cl^−^, NO_3_^−^, SO_4_^2−^, Na^+^, NH_4_^+^, K^+^, Mg^2+^, and Ca^2+^) levels in the four seasons was winter > autumn > spring > summer. This seasonal pattern is consistent with the seasonal distributions of outdoor PM_2.5_ concentrations, and that commonly observed in other cities in China [13]. The total concentrations of the ions were 23.14 μg/m^3^, 15.87 μg/m^3^, 28.82 μg/m^3^, and 33.11 μg/m^3^ in spring, summer, autumn, and winter, respectively. Compared with the results from other studies, the concentrations of the ions observed in this study were similar to the results from another study conducted in Nanjing [8], but much lower than in some heavily polluted cities in China, such as Tianjin [41], Jinan [25], and Beijing [32]. 

In indoor PM_2.5_, the concentrations of the ions were 12.18 μg/m^3^, 17.74 μg/m^3^, 25.40 μg/m^3^, and 27.04 μg/m^3^ in spring, summer, autumn, and winter, respectively. Generally, the total concentration of water-soluble ions in the indoor PM_2.5_ was lower than that from outdoors except in summer. Different seasonal variations of the water-soluble ions in indoor PM_2.5_ were observed in samples from the outdoors, in the order of winter > autumn > summer > spring (Figure 1). The highest indoor concentration of ions was observed in winter, similar to that in the outdoor PM_2.5_. However, the average concentration of the investigated ions in summer was higher than that in spring. In summer, windows were often closed while air conditioning was used due to the high temperature in Nanjing [36], which may lead to the accumulation of PM_2.5_ and water-soluble ions in the summer. The average concentrations of water-soluble ions in this study are either higher [42] or lower [43,44] than those of other studies conducted in a single season, mainly depending on the concentration of ions in the outdoor PM_2.5_ and indoor activities [25,41].

Due to the differences in the sampling households in different seasons, the seasonal trends of water-soluble ions in homes sampled across the four seasons (*N* = 11) were further analyzed. For the outdoor PM_2.5_ samples, the average concentrations of the eight ions were 13.56 μg/m^3^, 11.18 μg/m^3^, 21.19 μg/m^3^, and 23.54 μg/m^3^ in spring, summer, autumn, and winter, respectively. Whereas in indoor environments, the concentrations were 8.50 μg/m^3^, 11.57 μg/m^3^, 20.19 μg/m^3^, and 18.57 μg/m^3^ in spring, summer, autumn, and winter, respectively. The overall seasonal trends of water-soluble ions in the 11 homes are similar to that of the total samples (Figure 1), indicating that the differences between sampling homes across the four seasons did not affect the seasonal variations of heavy metals in indoor and outdoor PM_2.5_.

To better understand the difference in the water-soluble ions between the indoor and outdoor environment, the mass proportions of the water-soluble ions were calculated by dividing the mass concentrations of ions by the corresponding PM_2.5_ mass concentrations. For the outdoor samples, the investigated water-soluble ions accounted for approximately 18.99%, 59.29%, 46.10%, and 43.67% of the outdoor PM_2.5_ mass in spring, summer, autumn, and winter, respectively. In the residential indoor environment, the water-soluble ions accounted for approximately 19.71%, 46.38%, 48.53%, and 44.81% of the indoor PM_2.5_ mass in spring, summer, autumn, and winter, respectively. The highest mass proportions of water-soluble ions were observed in summer for both indoor and outdoor samples, followed by autumn, winter, and spring, which is different from the seasonal trends of the PM_2.5_ concentrations. The result indicated that the higher PM_2.5_ concentrations in winter and autumn did not increase the proportion of water-soluble ions.

Secondary inorganic aerosols (NO_3_^−^, SO_4_^2−^, and NH_4_^+^) were found to be the dominant constituents for both indoor and outdoor PM_2.5_, accounting for approximately 64.22%–88.07% and 78.73%–87.41% of the mass of water-soluble ions in indoor and outdoor PM_2.5_, respectively. The result is consistent with the studies conducted by Wang et al. [41] and He et al. [14]. SO_4_^2−^, NO_3_^−^, and NH_4_^+^ were formed through photochemical reactions with SO_2_, NO_2_, and NH_3_ [25,45]. The contributions of sea-salt species (Cl^−^ and Na^+^) and crustal elements (K^+^, Ca^2+^, Mg^2+^) to the total water-soluble ions were observed to be much lower than the total secondary aerosols in indoor and outdoor PM_2.5_. Hassanvand et al. investigated the water-soluble concentrations in particles of a different size and found that sea-salt species and crustal elements were mainly found in coarse particles [42].

#### 3.1.3. Metallic Elements in PM_2.5_

During the sampling period, the average concentrations of the total metallic elements (Pb, Mn, Cu, Cr, As, and Cd) were 209.78 ± 196.18 ng/m^3^ and 221.58 ± 163.49 ng/m^3^ in indoor and outdoor environments, respectively. Indoor concentrations of total elements were slightly lower than those from the outdoors, indicating that indoor environments were effective at providing protection from metallic element pollution in outdoor PM_2.5_. 

**Figure 2:**
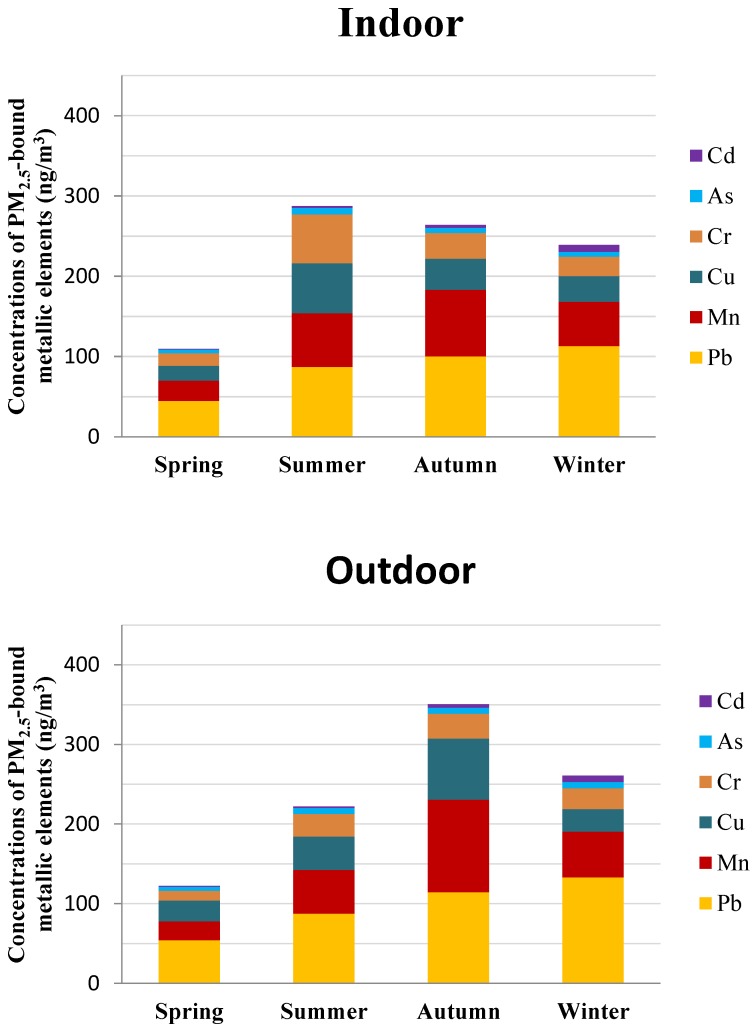
Seasonal variations of metallic elements in indoor and outdoor PM_2.5_.

Both the indoor and outdoor mass concentrations of the metallic elements varied with the season significantly (Figure 2). The total concentration of metallic elements in outdoor environments was highest in autumn (349.01 ± 257.15 ng/m^3^), followed by winter (259.14 ± 151.62 ng/m^3^), summer (221.96 ± 81.07 ng/m^3^), and spring (100.44 ± 64.47 ng/m^3^). Whereas in indoor environments, the highest metallic elements concentrations occurred in summer (287.12 ± 301.72 ng/m^3^), followed by autumn (262.20 ± 129.68 ng/m^3^), winter (236.05 ± 98.81 ng/m^3^), and spring (72.30 ± 41.28 ng/m^3^). The seasonal trends of metallic elements’ concentrations in the 11 homes that participated across the four seasons were also analyzed. The average concentrations of the metallic elements in outdoor PM_2.5_ were 84.92 ng/m^3^, 195.44 ng/m^3^, 270.49 ng/m^3^, and 229.10 ng/m^3^ in spring, summer, autumn, and winter, respectively. For indoor PM_2.5_ samples, the mean concentrations of the investigated metallic elements were 77.52 ng/m^3^ in spring, 221.65 ng/m^3^ in summer, 211.79 ng/m^3^ in autumn, and 201.52 ng/m^3^ in winter, respectively. The seasonal variations in indoor and outdoor PM_2.5_ samples in the 11 homes was consistent with the trends of the total samples, indicating that the difference of sampling homes across the four seasons did not affect the seasonal variations of metallic elements in indoor and outdoor PM_2.5_. The seasonal variations of indoor and outdoor PM_2.5_ concentrations was discussed in our previous study, with the highest concentrations occurring in winter and the lowest occurring in summer [36]. In this study, indoor and outdoor metallic elements exhibited a different seasonal trend from that of PM_2.5_ mass concentrations. Although the highest PM_2.5_ concentrations in winter were observed in the outdoor samples, the highest total metallic element concentrations were observed in autumn. In summer, PM_2.5_ concentrations were lowest in indoor environments. However, the metallic element concentration was observed to be the highest. The results indicated that the lower concentrations of PM_2.5_ may not result in the lower concentrations of metallic elements. In addition to the seasonal variations of PM_2.5_ concentrations, more attention should be paid to its chemical composition, such as the metallic elements, which are harmful to humans.

The mass proportions of the metallic elements in indoor and outdoor PM_2.5_ were also calculated by dividing the mass concentrations of the investigated elements by the corresponding PM_2.5_ mass concentrations. Metallic elements accounted for approximately 0.18%, 0.97%, 0.78%, and 0.51% of the outdoor PM_2.5_ samples in spring, summer, autumn, and winter, respectively. For indoor samples, the investigated elements accounted for 0.15%, 0.94%, 0.59%, and 0.54% of the indoor PM_2.5_ mass in spring, summer, autumn, and winter, respectively. The seasonal variation of the mass proportions of the metallic elements was consistent with that of the water-soluble ions. However, the mass proportions of metallic elements in PM_2.5_ were significantly lower than the ions. Moreover, the mass proportions of elements in indoor PM_2.5_ were slightly lower than that of the outdoor PM_2.5_. Indoor PM_2.5_ comes from both indoor and outdoor sources, and the lower proportions of metallic elements in indoor PM_2.5_ indicate that the content of metallic elements in PM_2.5_ generated by indoor sources may be lower than that of the outdoor sources.

Of the individual metals, the mass concentrations of the metallic elements in indoor PM_2.5_ decreases in the order as follows: Pb > Mn > Cu > Cr > As > Cd in spring; Pb > Mn > Cu > Cr > As > Cd in summer; Pb > Mn > Cu > Cr > As > Cd in autumn; and Pb > Mn > Cu > Cr > Cd > As in winter. Similar orders of elements were also found in the outdoor PM_2.5_, in the order of Pb > Cu > Mn > Cr > As > Cd in spring; Pb > Mn > Cu > Cr > As > Cd in summer; Pb > Mn > Cu > Cr > As > Cd in autumn; and Pb > Mn > Cu > Cr > Cd > As in winter. Pb, Mn, and Cu were the dominant components of the metallic elements in the indoor PM_2.5_ in the four seasons, accounting for approximately 81.53%–89.95% of the total metallic elements in the PM_2.5_, which is consistent with that from the outdoors (82.76%–90.60%). Specially, Pb was the most abundant element and the only element that exhibited lower concentrations in indoor samples than outdoor samples over the four seasons, indicating that outdoor sources were the main contributor to indoor Pb in PM_2.5_. The pollution of particle-associated Pb is widespread in China due to the use of leaded gasoline, although it has been prohibited since 2000 [15]. Pb concentration in the troposphere has decreased as a consequence of the phasing-out of leaded gasoline, but Pb remains essentially affected by anthropogenic sources in atmospheric aerosols [46]. Motor vehicle exhaust was considered to be the main source of Pb emissions [47]. The highest concentrations of Pb in indoor and outdoor PM_2.5_ samples were also found by Bi et al. in Nanjing [28]. As and Cd were found to have the lowest concentrations in this study. As and Cd are indicators of particulate matter from coal burning [33]. Zhang et al. found that significant higher concentrations of As and Cd were in seasons where heating was used compared to seasons without heating use [26]. The concentrations of As and Cd were low in this study, indicating a lower influence of coal burning sources on PM_2.5_ samples.

### 3.2. Relationship of Indoor and Outdoor PM_2.5_ Chemical Composition

#### 3.2.1. Water-Soluble Ions

The I/O ratios and indoor-outdoor Spearman correlation coefficients (r) for water-soluble ions in PM_2.5_ were calculated and are presented in Table 3. 

The I/O ratio is an indicator for evaluating the difference between indoor concentrations and the corresponding outdoor levels or for evaluating the strength of indoor/outdoor sources [48]. In this study, higher I/O ratios of water-soluble ions in summer and winter were found, while the I/O ratios in spring and autumn were much lower. The results suggested that there were different sources for water-soluble ions in different seasons. In hot summers and cold winters, windows were often closed by occupants while air conditioners were used [36], which may reduce indoor ventilation and result in the accumulation of indoor water-soluble ions. In spring and autumn, most of the I/O ratios of ions were less than 1, suggesting that the indoor ions were generated by outdoor sources. The temperature is favorable in spring and autumn and the windows were often opened for ventilation, which can increase the indoor air-exchange rate and outdoor air pollution infiltration [48].

The correlation between indoor and outdoor ions in PM_2.5_ in different seasons is shown in Table 3. Results showed that most of the indoor and outdoor water-soluble ions in PM_2.5_ were significantly correlated (*p* < 0.1) except Mg^2+^ and Ca^2+^. Crustal elements (Mg^2+^ and Ca^2+^) mainly come from outdoor sources, such as dust from soil, road surfaces, and construction, and they often exist in coarse particles, such as PM_10_ [42]. Moreover, the lower detection rate of the two cations (indoor Mg^2+^: 14.41%; Ca^2+^: 55.08% vs. outdoor Mg^2+^: 15.25%; Ca^2+^: 60.17%) in this study may also lead to the lack of an indoor-outdoor correlation for the two ions. The correlation coefficients (r) between indoor and outdoor ions were observed to be higher in transitional seasons (spring and autumn) than in summer and winter due to the long duration of ventilation in transition seasons. The result is consistent with the results of the I/O ratios of the ions.

#### 3.2.2. Metallic Elements

The indoor to outdoor (I/O) ratios and indoor-outdoor Spearman correlation coefficients for metallic elements in PM_2.5_ are presented in Table 4. 

The I/O ratios in summer and winter are above 1 for all elements. In spring and autumn, relatively lower I/O ratios for most of the elements were observed. The seasonal variations of I/O ratios for metallic elements are consistent with that of the water-soluble ions in PM_2.5_ discussed in Section 3.2.1. The difference in indoor ventilation may be the main reason for the seasonal variations of the elements’ I/O ratios. Some individual elements exhibited a different seasonal pattern from the others, such as Cr and Cd. For the elements, Cr and Cd, higher I/O ratios can still be found in spring and autumn than those of other elements, suggesting that there may be indoor sources for the two elements. Cr and Cd are mainly derived from indoor sources, such as cigarette smoke, cosmetics, and paint chips [27,49]; this results in higher I/O ratios for these two elements.

The results of the Spearman correlation analysis showed that Pb, Mn, As, and Cd in the indoor and outdoor PM_2.5_ were significantly correlated in most seasons (*p* < 0.05). The results indicated that outdoor concentrations were the main factors determining the indoor concentrations of Pb, Mn, As, and Cd. Indoor Cu and Cr concentrations were only observed to be correlated with those of the outdoors in transitional seasons (Cu in spring and Cr in autumn), suggesting the main contributions of indoor sources for Cu and Cr.

### 3.3. Health Risk Assessment of Metallic Elements

Toxic metallic elements in PM_2.5_ have potential effects on human health through inhalation, dermal contact, and ingestion into human bodies [50], especially for children who are more sensitive to these metals than adults due to their still developing immune system and lungs [51], their age, lower body weight, and lower tolerance to toxins [52]. In this study, a health risk assessment of metallic elements in PM_2.5_ through inhalation pathways was conducted for both adults and children. 

The carcinogenic risks for metallic elements from indoor and outdoor PM_2.5_ are shown in Table 5. The risk levels of carcinogenic metals for indoor and outdoor PM_2.5_ were all within the acceptable levels (between 1.0 × 10^−4^ and 1.0 × 10^−6^), and the risk of metals decreased in the following order: Cr > As > Cd > Pb. The higher concentration of PM_2.5_-bound Pb did not result in a greater carcinogenic risk due to the lower inhalation unit risk for Pb shown in Table 2. Cr and As were found to be the two highest cancer-risk carcinogenic elements in PM_2.5_. The research from Wang et al. [31] in Nanjing also revealed that As and Cr in residential and ambient environments represented the highest cancer-risk to people. Similar results can be found in Tianjin and Lanzhou, China, which indicated that Cr and As posed the highest carcinogenic risk [26,53]. The cancer risk of the metallic metals in the outdoor PM_2.5_ was observed to be higher than that in the indoor PM_2.5_ except for Cr, which exhibited a higher risk in indoor environments than outdoors. Cr is mainly derived from cigarette smoke, cosmetics, and paint chips [49]. In this study, indoor smoking activities may be the cause of higher indoor Cr concentrations and cancer risk [36]. Moreover, the investigated metallic elements posed a higher cancer risk to adults than children. The total cancer risk of metallic elements was higher than 10^−6^, but lower than 10^−4^, indicating that the cancer risk caused by metallic elements in indoor and outdoor PM_2.5_ in this study was acceptable.

The noncarcinogenic risk described in HQ for metallic elements is shown in Table 5. The HQ values of the four metallic elements occurred in the following order: Mn > Cr > As > Cd (indoor) and Mn > As > Cd > Cr (outdoor). For the indoor and outdoor samples, the HQ values of Mn were higher than the acceptable level of 1.0, while the HQ values of Cr, As, and Cd were all lower than 1. Our results indicate that Cr, As, and Cd in PM_2.5_ would not result in noncarcinogenic health effects while Mn may cause adverse health effects. The higher noncarcinogenic risk of Mn was also found in other studies [26,53]. The HQ value of Mn in indoor environments was higher than the results conducted by Wang et al. in Nanjing, in which Mn also exhibited the highest noncarcinogenic risk over the other heavy metals [31]. Mn is used in unleaded gasoline as an anti-knocking agent and can also be used as an additive in petroleum products [54]. Moreover, Mn in PM_2.5_ had significant natural source characteristics and may be derived from soil dust [31]. As shown in Table 1, the sampling homes in this study were mostly near the roadside, so the indoor and outdoor air may be polluted by exhaust from vehicles and soil dust, resulting in the higher HQ values of Mn. According to Equation (2), the exposure concentration of metals in PM_2.5_ is equal to the contaminant concentration in the air for the noncarcinogenic risk assessment, thus there were no differences in the calculated noncarcinogenic risk between adults and children in this study. Although the HQ value of most individual metallic elements was below 1, except for Mn, the total noncarcinogenic risk HI value was higher than 1. The results suggested that the accumulative risk of exposure by inhalation to noncarcinogenic PM_2.5_-bound metallic elements was important to the health risk assessment. The HI value of all metallic elements was more than two times higher than safe levels, indicating that the total metallic elements in PM_2.5_ can cause potential adverse health effects for residents. Therefore, more attention should be paid to inhalation exposure of PM_2.5_ with toxic metallic elements.

## 4. Conclusions

This study investigated the concentrations of chemical components in indoor and outdoor PM_2.5_ in residences in Nanjing, China. Different seasonal variations were observed for water-soluble ions and metallic elements in indoor and outdoor PM_2.5_. The highest water-soluble ions in outdoor PM_2.5_ samples occurred in winter, and the lowest occurred in summer, which is consistent with the seasonal variations of outdoor PM_2.5_ concentrations. Water-soluble ions in indoor PM_2.5_ samples varied with the outdoor levels, except in summer, when higher concentrations of ions in indoor environments were observed compared to the outdoors. When indoor ventilation was lacking, especially in cold winters and hot summers, higher I/O ratios and lower indoor-outdoor correlations of water-soluble ions in PM_2.5_ were observed, suggesting the influence of indoor sources in the two seasons. Secondary inorganic aerosols (NO_3_^−^, SO_4_^2−^, and NH_4_^+^) were the dominant constituents of water-soluble ions in both indoor and outdoor PM_2.5_, which also exhibited higher indoor-outdoor correlations than other ions; this indicates that indoor secondary inorganic aerosols mainly come from the outdoors. 

The metallic elements in indoor PM_2.5_ exhibited different seasonal variations compared to the outdoors. The highest concentrations in indoor PM_2.5_ were found in summer, followed by autumn, winter, and spring; for the outdoor samples, the highest concentrations occurred in autumn, followed by winter, summer, and spring. The difference in seasonal variations of metallic elements between indoor and outdoor PM_2.5_ indicated that even when the outdoor concentrations of metallic elements was low, people may also be exposed to higher concentrations of elements due to the accumulation of air pollutants in indoor environments. To reduce the exposure to metallic elements indoors, people should consider both indoor and outdoor sources comprehensively. The I/O ratios of metallic elements were higher than that of water-soluble ions, and the correlation coefficients were much lower. The results indicated that there may be different sources for indoor water-soluble ions and metallic elements. Further research should be conducted to investigate the source apportionment of indoor chemical compositions and provide more suggestions for improving indoor air quality and reducing human exposure and health impacts.

The health risk of metallic elements in indoor and outdoor PM_2.5_ was calculated in this study. A lower health risk for indoor metallic elements than the outdoors was observed except for Cr, suggesting that there is a significant influence of indoor sources for Cr in indoor environments. The metallic elements posed a higher health risk to adults than children. The risk levels of carcinogenic metals for indoor and outdoor PM_2.5_ were all within the acceptable levels. The non-carcinogenic risks of most of the elements were within the safe limits. Mn was found to have a non-carcinogenic risk to residents in Nanjing, and the exhaust from vehicles and soil dust may be the potential sources of Mn in indoor and outdoor PM_2.5_. More attention should be paid to the pollution of Mn in PM_2.5_ in Nanjing to reduce its health impacts on residents. Moreover, a cumulative effect of noncarcinogenic risk for the elements was found to be more than two times higher than the safe levels, indicating that more attention should be paid to the inhalation exposure of PM_2.5_ with toxic metallic elements.

## Figures and Tables

**Table 1:** Descriptions of home characteristics.

Variables		Spring *N* (%)	Summer *N* (%)	Autumn *N* (%)	Winter *N* (%)
Building Height	Multi-story	18 (52.9)	16 (45.7)	11 (55.0)	20 (57.1)
	High-rise	16 (47.1)	19 (54.3)	9 (45.0)	15 (42.9)
Construction date	Before 1990	6 (17.6)	6 (17.1)	3 (15.0)	5 (14.3)
	1991–2000	5 (14.7)	4 (11.4)	5 (25.0)	9 (25.7)
	2001–2010	5 (14.7)	7 (20.0)	5 (25.0)	10 (28.6)
	After 2011	18 (52.9)	18 (51.4)	7 (35.0)	11 (31.4)
Floor area	<60 m^2^	5 (14.7)	7 (20.0)	3 (15.0)	9 (25.7)
	60–90 m^2^	15 (44.1)	13 (37.1)	8 (40.0)	16 (45.7)
	90–120 m^2^	11 (32,4)	12 (34.3)	7 (35.0)	6 (17.1)
	>120 m^2^	3 (8.8)	3 (8.6)	2 (10.0)	4 (11.4)
Building orientation	North-South	21 (61.8)	25 (71.4)	12 (60.0)	24 (68.6)
	Other	13 (38.2)	10 (28.6)	8 (40.0)	11 (31.4)
Distance to the main traffic Road	<100 m	12 (35.3)	16 (45.7)	13 (65.0)	15 (42.9)
	100–500 m	13 (38.2)	13 (37.1)	5 (25.0)	13 (37.1)
	500–1000 m	9 (26.5)	6 (17.1)	2 (10.0)	6 (17.1)
Dwelling ownership	Owner	28 (82.4)	28 (80.0)	13 (65.0)	30 (85.7)
	Tenant	6 (17.6)	7 (20.0)	7 (35.0)	5 (14.3)
Number of occupants	≤1	2(5.9)	1 (2.90)	1 (5.0)	2 (5.7)
	2–3	15 (44.1)	16 (45.7)	6 (30.0)	14 (40.0)
	4–5	17 (50.0)	17 (48.6)	13 (65.0)	18 (51.4)
	≥6	-	1 (2.9)	-	1 (2.9)

**Table 2:** Relative toxicity values of metallic elements used in this study.

Toxicity Parameters	Pb	Mn	Cu	Cr	As	Cd
IUR (μg/m^3^))^−1^	1.2 × 10^−5^	-	-	1.2 × 10^−2^	4.3 × 10^−3^	1.8 × 10^−3^
RfC (mg/m^3^)	-	5.0 × 10^−5^	-	1.0 × 10^−4^	1.5 × 10^−5^	1.0 × 10^−5^

**Table 3:** The indoor–outdoor relationship of water-soluble ions in PM_2.5_.

Ion	Spring		Summer		Autumn		Winter	
	I/O	*r*	I/O	*r*	I/O	*r*	I/O	*r*
Cl^−^	0.96 ± 1.04	**0.88 ^b^**	1.19 ± 0.57	**0.60 ^b^**	0.94 ± 0.18	**0.68 ^a^**	0.89 ± 0.22	**0.46 ^a^**
NO_3_^−^	0.72 ± 0.51	**0.69 ^b^**	1.29 ± 0.57	**0.61 ^b^**	0.92 ± 0.35	**0.68 ^a^**	0.89 ± 0.50	**0.62 ^b^**
SO_4_^2−^	0.98 ± 0.15	**0.71 ^b^**	1.12 ± 0.66	**0.60 ^b^**	0.99 ± 0.32	**0.66 ^a^**	0.90 ± 0.39	**0.61 ^a^**
Na^+^	1.27 ± 1.29	**0.80 ^b^**	1.26 ± 0.91	**0.50 ^a^**	0.64 ± 0.51	0.22	4.67 ± 8.55	0.04
NH_4_^+^	0.66 ± 0.35	**0.84 ^b^**	3.05 ± 5.06	**0.93 ^b^**	0.55 ± 0.40	**0.91 ^b^**	1.45 ± 2.49	**0.63 ^b^**
K^+^	0.90 ± 0.97	**0.65 ^b^**	1.73 ± 2.64	**0.56 ^a^**	0.74 ± 0.38	**0.89 ^b^**	1.40 ± 1.94	0.09
Mg^2+^	0.87 ± 0.66	**0.51 ^a^**	-	0.06	-	-	-	-
Ca^2+^	1.85 ± 2.02	0.27	-	0.07	0.63 ± 0.40	**0.80 ^c^**	1.29 ± 1.29	0.02

^a^*p* < 0.01; ^b^ *p* < 0.001; - lack of data due to the low detection rate of water-soluble ions.

**Table 4:** The indoor–outdoor relationship of metallic metals in PM_2.5_.

Element	Spring		Summer		Autumn		Winter	
	I/O	r	I/O	r	I/O	r	I/O	r
Pb	0.90 ± 0.35	**0.62 ^c^**	1.07 ± 0.33	**0.50 ^b^**	0.92 ± 0.41	0.44	1.01 ± 0.50	**0.58 ^c^**
Mn	0.55 ± 0.35	**0.40 ^a^**	1.26 ± 0.87	**0.39 ^a^**	0.84 ± 0.37	**0.61 ^b^**	1.34 ± 1.67	**0.43 ^b^**
Cu	0.80 ± 0.55	**0.62 ^c^**	2.37 ± 5.43	0.28	0.86 ± 0.64	0.00	7.92 ± 23.37	0.21
Cr	0.89 ± 0.43	0.20	2.65 ± 7.89	0.02	1.49 ± 1.02	**0.49 ^a^**	1.18 ± 0.82	0.08
As	0.88 ± 0.48	**0.78 ^c^**	1.21 ± 0.55	**0.82 ^c^**	0.96 ± 0.55	0.42	1.01 ± 0.58	**0.64 ^c^**
Cd	1.02 ± 0.62	**0.65 ^c^**	1.50 ± 1.14	**0.57 ^c^**	1.50 ± 1.64	**0.66 ^c^**	2.09 ± 3.01	**0.50 ^b^**

^a^*p* < 0.05; ^b^ *p* < 0.01; ^c^ *p* < 0.001.

**Table 5:** Carcinogenic and noncarcinogenic risks for metallic elements in indoor and outdoor PM_2.5_.

Metallic Elements	Indoor				Outdoor			
	Noncarcinogenic Risk (HQ)		Carcinogenic Risk (CR)		Noncarcinogenic Risk (HQ)		Carcinogenic Risk (CR)	
	Adult	Children	Adult	Children	Adult	Children	Adult	Children
Pb	-	-	2.58× 10^−^^6^	6.44× 10^−^^7^	-	-	2.94 × 10^−^^6^	7.35 × 10^−^^7^
Mn	1.15	1.15	-	-	1.30	1.30	-	-
Cr	4.80 × 10^−^^1^	4.80 × 10^−^^1^	2.82 × 10^−^^5^	7.06 × 10^−^^6^	2.58 × 10^−^^1^	2.58 × 10^−^^1^	1.51 × 10^−^^5^	3.79 × 10^−^^6^
As	4.69 × 10^−^^1^	4.69 × 10^−^^1^	1.04 × 10^−^^5^	2.59 × 10^−^^6^	5.31 × 10^−^^1^	5.31 × 10^−^^1^	1.18 × 10^−^^5^	2.94 × 10^−^^6^
Cd	4.64 × 10^−^^1^	4.64 × 10^−^^1^	2.86 × 10^−^^6^	7.16 × 10^−^^7^	4.89 × 10^−^^1^	4.89 × 10^−^^1^	3.02 × 10^−^^6^	7.54 × 10^−^^7^
Total	2.08	2.08	4.40 × 10^−^^5^	1.10 × 10^−^^5^	2.58	2.58	3.29 × 10^−^^5^	8.22 × 10^−^^6^
